# New Diagnosis of Obstructive Sleep Apnea Following Anterior Cervical Discectomy Complicated by Rheumatoid Arthritis: A Case Report

**DOI:** 10.7759/cureus.7439

**Published:** 2020-03-27

**Authors:** Waiz Wasey, Emir Festic, Amit Sapra, Taaha Rafi, Priyanka Bhandari

**Affiliations:** 1 Family and Community Medicine, Southern Illinois University School of Medicine, Springfield, USA; 2 Pulmonary Medicine, Mayo Clinic, Jacksonville, USA; 3 Family Medicine, Southern Illinois University School of Medicine, Springfield, USA

**Keywords:** anterior cervical discectomy and fusion, rheumatoid arthritis, radiculopathy, myelopathy, neck pain, snoring, risk factors for obstructive sleep apnea (osa), obstructive sleep apnea, worsening obstructive sleep apnea (osa)

## Abstract

Obstructive sleep apnea (OSA) is the most common variant of sleep-disordered breathing that often goes undiagnosed. OSA is characterized mainly by anatomical obstruction or partial collapse of upper airways during sleep. The obstruction is multifactorial, and a lesser-known fact is that damage to the pharyngeal plexus during head and neck procedures or placement of hardware in the cervical area can lead to narrowing or collapse of the upper airway. We present such a case of a 59-year-old female who developed new-onset OSA after undergoing anterior cervical discectomy and fusion (ACDF). The severity of OSA worsened with the progression of her rheumatoid arthritis (RA) in the cervical region. This case report aims to raise awareness of such an association among clinicians to enable them to screen appropriate patients for sleep-disordered breathing and treat them accordingly.

## Introduction

The onset of obstructive sleep apnea following anterior cervical discectomy and fusion has been published in a retrospective study of 12 patients [1]. Association of obstructive, as well as central sleep apnea with rheumatoid arthritis affecting the cervical vertebrae, has also been observed [1]. Herein, we present a case of a new diagnosis of obstructive sleep apnea (OSA) following anterior cervical discectomy and fusion (ACDF) with subsequent worsening caused by rheumatoid arthritis (RA), requiring further instrumentalization affecting the cervical spine.

## Case presentation

**Figure 1:**
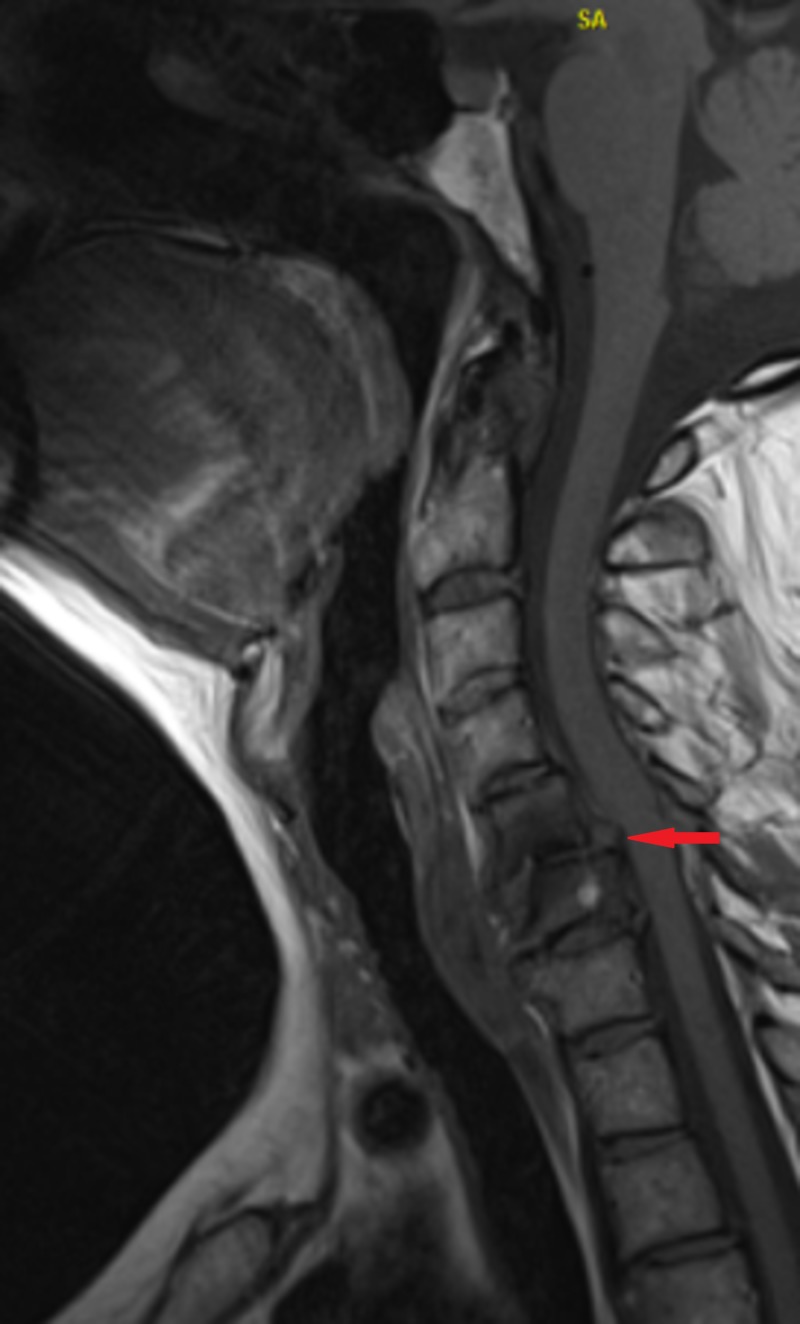
Magnetic resonance imaging of the cervical spine showing evidence of lower cervical spondylosis

A 59-year-old female with a history of hypertension, hyperlipidemia, and rheumatoid arthritis had been developing a progressive increase in pain, numbness, and loss of grip strength in the right upper extremity. She had no prior diagnosis of OSA or prior polysomnography. Magnetic resonance imaging (MRI) of the cervical spine showed prominent lower cervical spondylosis, most marked at cervical vertebrae C5-6, with mild to moderate central spinal stenosis and cord compression (Figure 1). 

**Figure 2:**
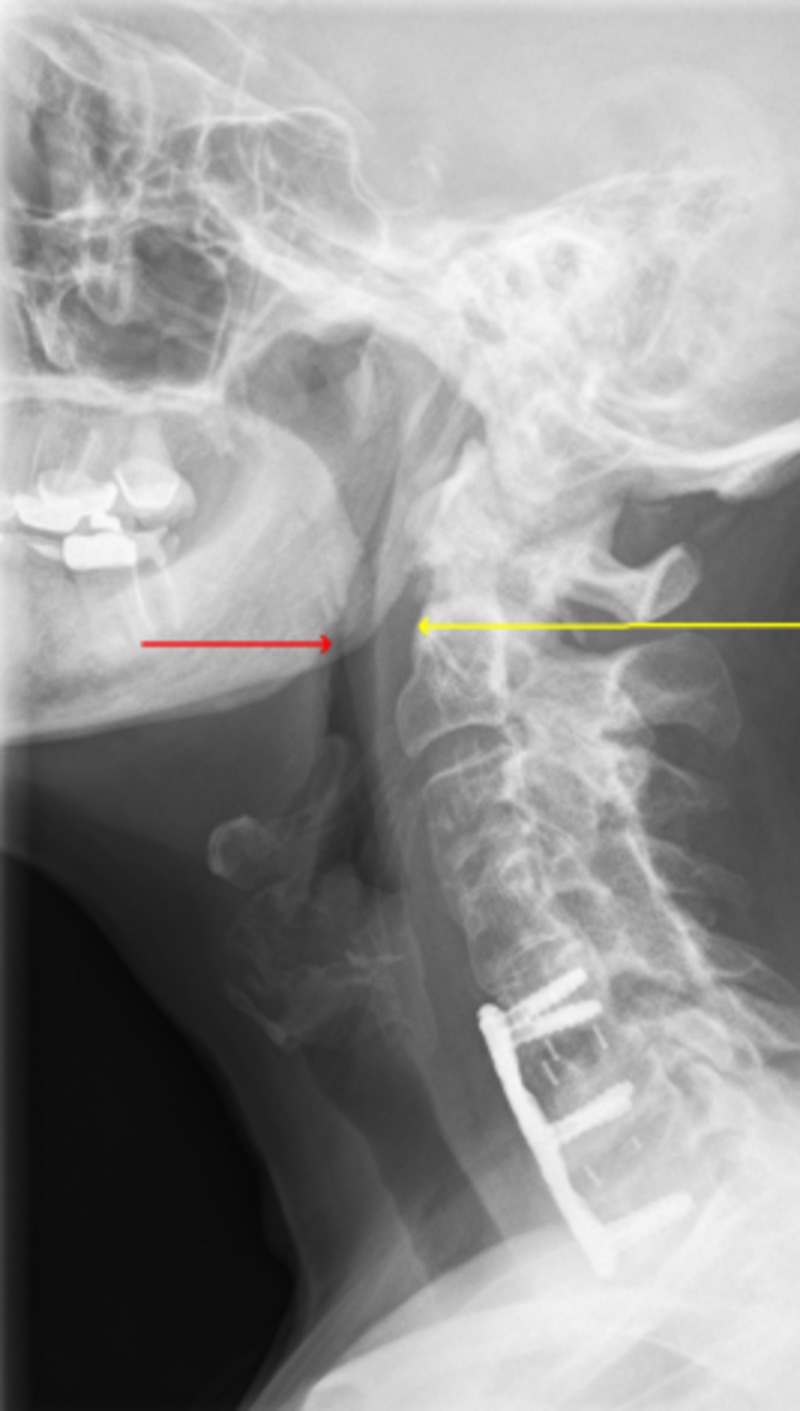
Postoperative cervical x-ray showing anterior plate and screw fixation at C5-7 with a narrowing airway (red and yellow arrows)

After evaluation with neurosurgery, she underwent ACDF of C5-6 and C6-7 with the placement of Synthes Vectra system anterior cervical plating (DePuy Synthes GmbH, Oberdorf, Switzerland) in C5-7. She was noted to have apneic episodes post-surgically in recovery. A postoperative x-ray of the cervical spine showed an anterior plate and screw fixation in C5-7 with no dynamic instability and very minimal motion with flexion and extension (Figure 2). 

**Figure 3:**
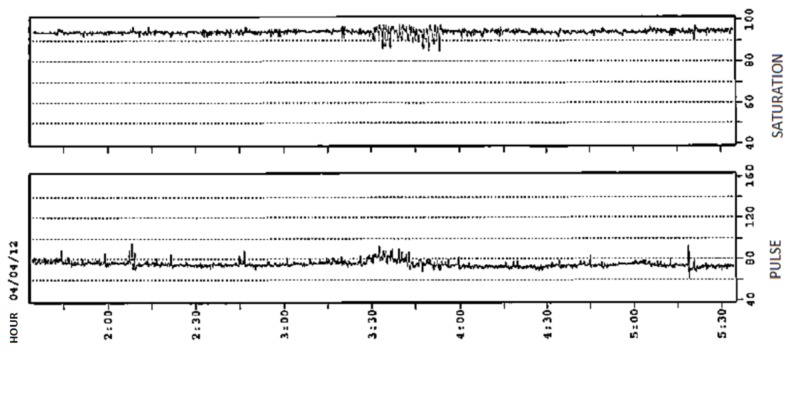
Oximetry showing a mild obstructive sleep pattern

Following discharge, she had overnight oximetry which showed a mild obstructive sleep apnea pattern (Figure 3).

She was then sent for a sleep study consultation. Following the evaluation, she underwent in-lab polysomnography (PSG) (Table 1).

**Table 1:** Polysomnography in June of 2012 O_2_: oxygen; PLMI: periodic leg movement index

Polysomnography - June 2012	
Total sleep time	415 minutes
Apnea/hypopnea Index	Supine: 40.5; Off back: 12.5
Mean O_2_	95.6%
PLMI	0.0/hour

**Figure 4:**
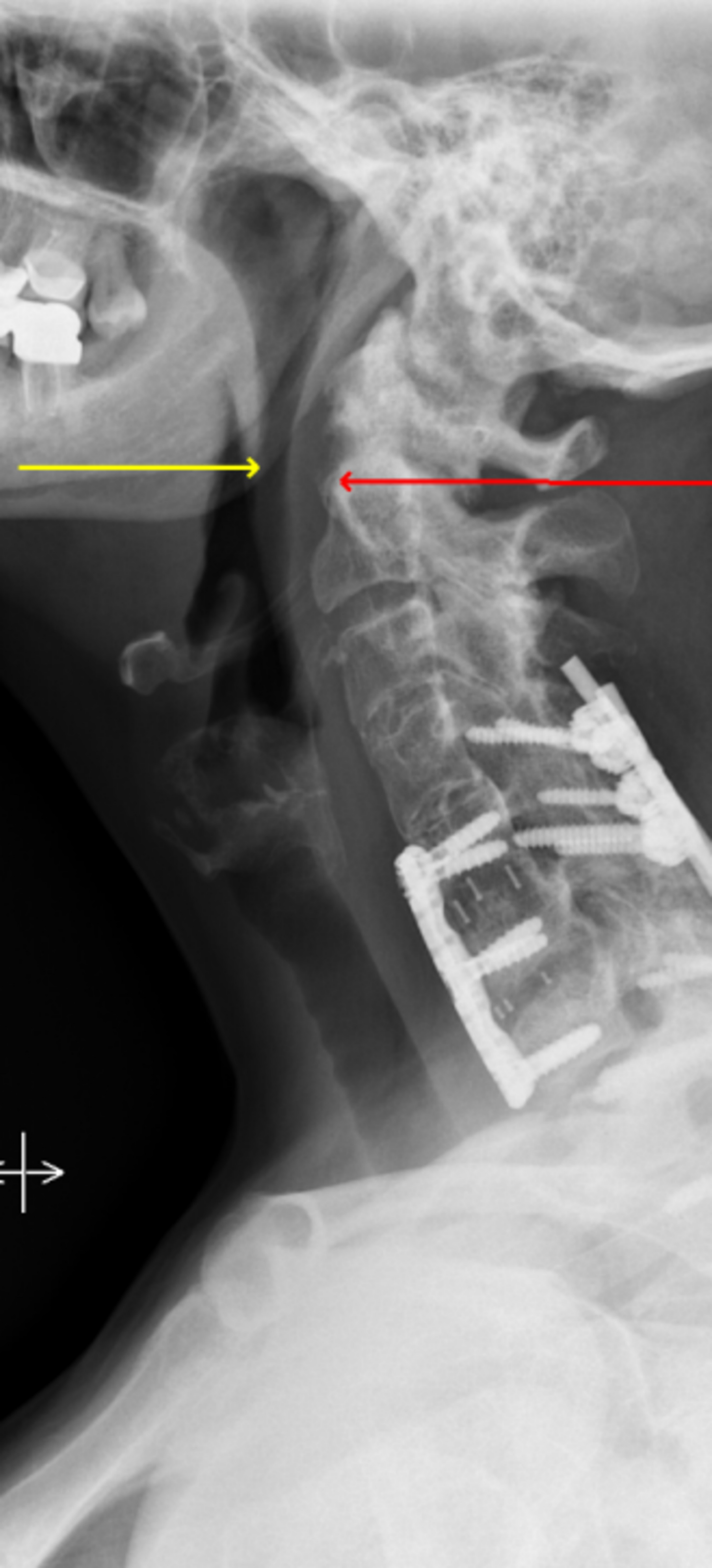
C4-T2 posterior instrumented fixation using the K2M Caspian system with further narrowing of airway (red and yellow arrows)

A diagnosis of mild obstructive sleep apnea was made and she was placed on continuous positive airway pressure (CPAP) at a setting of 8 cm H2O following a titration study in the sleep lab. Two years later her spinal recovery was complicated with worsening neck pain and numbness in the right upper extremity. Re-evaluation with imaging revealed pseudo-arthrosis at C6-7 as well as auto-fusion from her rheumatoid arthritis at C3-5. She underwent right C6-7 decompressive foraminotomy as well as C4-T2 posterior instrumented fixation using the K2M Caspian® system (Stryker Corp., Kalamazoo, MI) (Figure 4).

**Figure 5:**
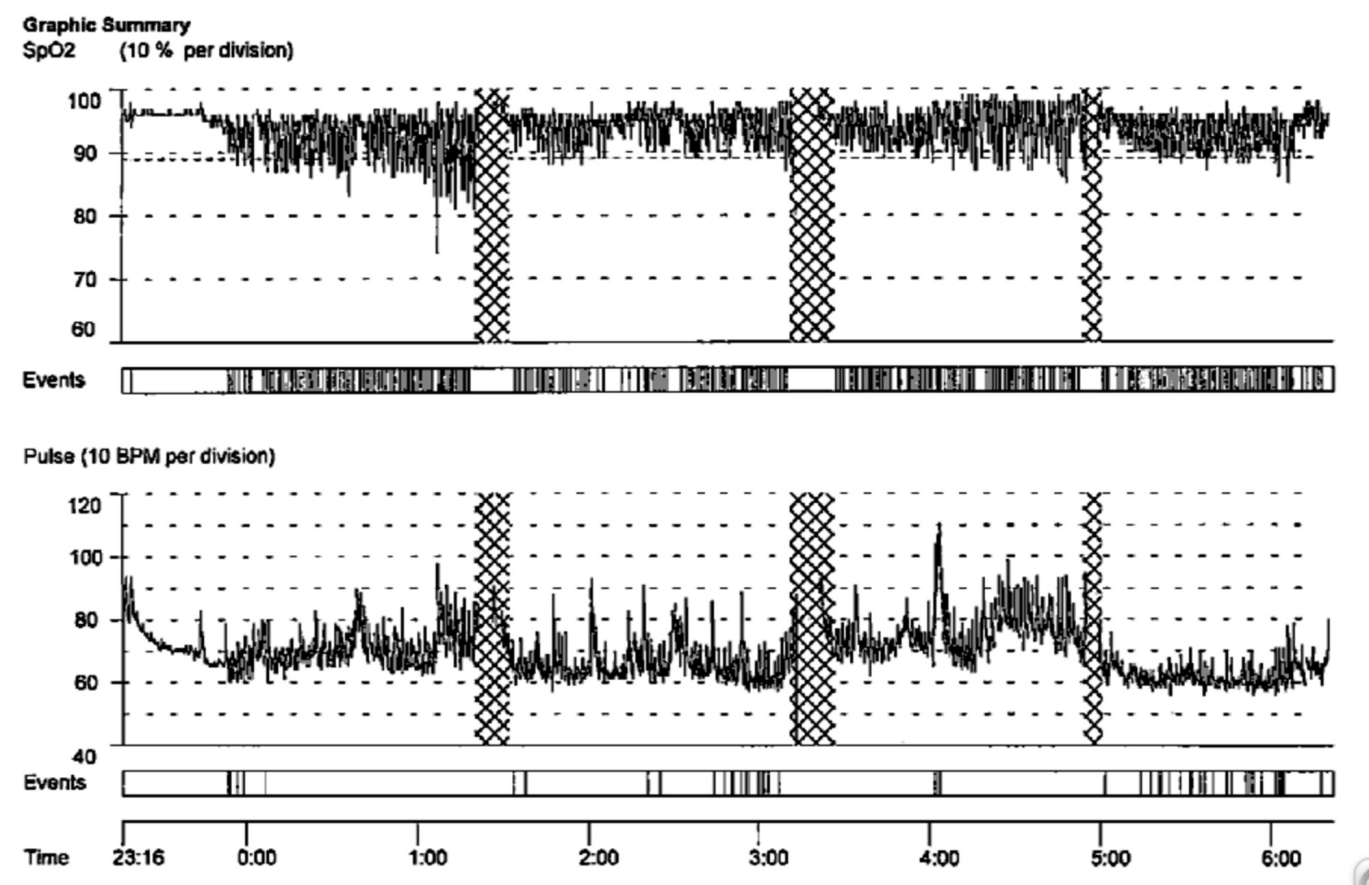
Oximetry showing severe obstructive sleep apnea pattern BPM: beats per minute; SPO_2_: saturation percentage of oxygen in the blood

On her follow-up visit with sleep medicine almost five years later, her husband reported that her OSA had worsened with increased snoring and increased apneic episodes. Her Epworth Sleepiness Scale at that visit was 5/24. She had also lost 20 - 30 pounds since her last PSG five years earlier. On examination, she did not appear to have a crowded oropharynx with a Friedman grade 1 palate. Oximetry was performed to reevaluate. Her oximetry this time showed a severe sleep-disordered breathing pattern (Figure 5).

Her PSG revealed moderate OSA (Table 2).

**Table 2:** Comparison of Polysomnography Done in June 2012 Versus November 2018 O_2_: oxygen; PLMI: periodic leg movement index

Polysomnography comparison	
June 2012	November 2018
Total sleep time (TST): 415 minutes	Total sleep time: 440 mins
Apnea/Hypopnea Index - 13.7; Supine - 40.5, Off back: 12.5	Apnea/Hypopnea Index - 24.7; Supine: 24.7; Off back: 24.7
Mean O_2_ - 95.6%	Mean O_2 _- 95.3%
PLMI - 0.0/hour	PLMI - 0.0/hour

## Discussion

ACDF is often used to treat patients with radiculopathy and myelopathy after the failure of non-operative treatment. A retrospective investigation of 12 patients who developed OSA following ACDF was performed by Guilleminault et al. [2]. The authors found that the placement of the anterior fixation device reduced the size of the middle pharyngeal space, which led to the development of OSA. There have been reports of injury to the superior laryngeal nerve leading to dysphagia [3]. The association of dysphagia with OSA in these patients may point towards a possibility of damage to the pharyngeal plexus during the surgery. A case report by Zhang and associates indicated the development of positional dyspnea in a patient who underwent ACDF, likely due to swelling in the middle pharyngeal space [4]. There is a need for a prospective investigation to establish an association between ACDF and OSA and to determine if it is a transient or long-term complication.

RA is a systemic inflammation afflicting multiple organ systems, particularly synovial joints. As the occiput-C1 and C1-C2 articulations are purely synovial, they are the primary targets for inflammation in RA. The association between RA and OSA was first described by Davies and associates [5]. Reduced neck width and decreased craniovertebral angles may crowd the retropharyngeal space, leading to a higher prevalence of OSA. In our case, the auto fusion of C3-C4 resulted in a narrowing of the airway and restriction of movement. Pepin and associates reported OSA due to subluxation of the cervical spine at the level of C3-C4 due to RA [6]. The association of reduced airway diameter due to temporomandibular joint regression as a result of RA and central sleep apnea due to compression of the medulla by the odontoid process have also been hypothesized from various case reports [1]. We acknowledge that part of the current study was published as a poster from the Sleep 2019 meeting, San Antonio, TX, June 10-13, 2019 [7].

## Conclusions

OSA is the most prevalent sleep-disordered breathing condition, as well as the most underdiagnosed. This case report aims to make the primary care physicians aware of an association between ACDF, RA, and OSA, with the aim that the patients undergoing such procedures are screened postoperatively for the possible development of OSA and treated accordingly. Prospective studies are required to follow ACDF patients to conclude that the OSA which develops in them is transient or permanent. The retrospective data available does point towards a strong association between ACDF and OSA. Treating such patients for sleep apnea will improve their quality of life and help them tolerate pain better.
